# The Cholera Scare

**Published:** 1883-11

**Authors:** Frank W. Reilly

**Affiliations:** Springfield, Ill.


ARTICLE V.

### THE CHOLERA SCARE.

The Chicago Daily News has been obtaining the views of physicians and sanitarians with reference to the probability of Asiatic cholera visiting this country next year, and their opinion as to what should be done by municipal authorities and households to avert an epidemic of that pestilence. The questions are of interest to our own citizens as well as to those of Chicago, and the replies may be summarily stated to be overwhelmingly in favor of the probability of the next summer witnessing an invasion of the dreaded disease, and of the urgent necessity of "cleaning up" in every possible way.

Among those whose views and advice were sought was Dr. Frank Reilly, of this city, the Assistant Secretary of the State Board of Health. The doctor declines to guess as to the question whether cholera will or will not come next year, but points out that the same conditions which would favor its spread, if it should come, cause every year an enormously larger mortality than epidemics themselves do. The following is the text of his letter:

MY DEAR SIR:—I am, this evening, in receipt of your circular of inquiry, forwarded to me from Chicago, and in which you ask a brief statement of my views "as to the probability that this country will suffer from the threatened epidemic" of Asiatic cholera, and as to the precautions which should be adopted, etc.

I am reconciled to the fact that my "views" concerning such a probability are worth no more than, if so much as, those of the editor of a metropolitan newspaper, with his facilities for judging of probabilities, by the other fact that epidemics are by no means unmixed evils. It was the recurrence of cholera epidemics which directly led to the first attempt at sewers in Chicago, and the present system of water supply. It was the recurrence of yellow fever epidemics in Memphis which led to the sanitary regeneration of that city. It was the recurrence of epidemics of both diseases which resulted in the magnificent sanitary work, educational and practical, of the New Orleans auxiliary sanitary association.

The salutary spur of an occasional epidemic outbreak seems to be necessary in order to secure any decent amount of attention to the care of the public health, at least while the sanitary schoolmaster is so much abroad as at present. In any prolonged exemptions from such visitations communities become lax, and gradually grow to tolerate conditions which result, directly or indirectly, in an enormously greater aggregate of mortality than that caused by an epidemic.

For example: The class of diseases to which Asiatic cholera belongs, and which are all more or less preventable, caused 5,136 deaths in an aggregate mortality of 13,324 in Chicago last year. In 1881 it caused 5,985 deaths out of a total of 14,101. This is an average of 40 per cent. of the total mortality. The last epidemic of cholera (1873) caused only 3,825 deaths in the whole country—nineteen States being invaded. There had been no epidemic cholera in the United States for six years previous, since 1866; but during those six years, not less than 125,000 persons had been carried off by the group of diseases most closely resembling it.

The moral of these figures seems to me no less obvious than that of the tables in the report of the Health Department, recently issued. Take from this report the table of zymotic mortality by wards and compute the ratio of this mortality to population. The result will furnish a very accurate index of the sanitary status of each ward. Where the zymotic mortality is greatest, there will be found the most overcrowding, the greatest amount of personal uncleanliness, the greatest want of sewerage, the most neglected scavenging, the most abundant and various filth, both surface and subterranean. Epidemic cholera might temporarily increase the death-rate under these conditions, but the average mortality for a series of years would not be materially affected.

Enough, however, in this strain. I suppose there is no prosing nor prophecy so insupportably dreary as that of the sanitary Cassandra. By way of amends, let me offer the following series of propositions concerning Asiatic cholera, formulated in a report which I drew up in 1875, at the request of Surgeon-General John M. Woodworth, of the marine hospital service, and under whose name it was published, in the volume entitled "The Cholera Epidemic of 1873 in the United States."—(Ex. Doc. No. 95, H. R. XLIIId Congress, 2d session.) These propositions are based upon a vast mass of cumulative evidence collected by cholera students in both hemispheres, and were originally intended to bear solely upon the question of the exclusion of the disease from this country. They will be found, however, equally applicable to methods of stamping out the disease should it effect foothold, and to the personal protection of the individual.

I. Malignant cholera is caused by the access of a specific organic poison to the alimentary canal; which poison is developed spontaneously only in certain parts of India (Hindostan).

II. This poison is contained primarily, so far as the world outside of Hindostan is concerned, in the vomit, stools, and urine of a person already infected with the disease.

III. To set up anew the action of the poison, a certain period of incubation, with the presence of alkaline moisture, is required, which period is completed within one to three days; a temperature favoring decomposition, and moisture or fluid of alkaline reaction, hastening the process, the reverse retarding.

IV. Favorable conditions for the growth of the poison are found (1) in ordinary potable water containing nitrogenous organic impurities, alkaline carbonates, etc.; (2) in decomposing animal and vegetable matter possessing an alkaline reaction; (3) in the alkaline contents of the intestinal portion of the alimentary canal.

V. The period of morbific activity of the poison—which lasts, under favorable conditions, about three days for a given crop—is characterized by the presence of bacteria, which appear at the end of the period of incubation and disappear at the end of the period of morbific activity. That is to say, a cholera-ejection, or material containing such, is harmless, both before the appearance and after the disappearance of bacteria, but is actively poisonous during their presence.

VI. The morbific properties of the poison may be preserved in posse for an indefinite period in cholera-ejections dried during the period of incubation, or of infection matter dried during the period of activity.

VII. The dried particles of cholera poison may be carried (in clothing, bedding, etc.) to any distance, and when liberated may find their way direct to the alimentary canal through the medium of the air—by entering the nose and mouth and being swallowed with the saliva—or, less directly, through the medium of water or food in which they have lodged.

VIII. The poison is destroyed naturally either by the process of growth or by contact with acids: (1) those contained in water or soil; (2) acid gases in the atmosphere; (3) the acid secretion of the stomach.

IX. It may be destroyed artificially: (1) by treating the cholera-ejections, or material containg them, with acids; (2) by such acid (gaseous) treatment of contaminated atmosphere; (3) by establishing an acid diathesis of the system in one who has received the poison.

For the non-professional reader the pith of these propositions is contained in the last two—the eighth and ninth. Nothing has since come under my observation to change the conviction arrived at, when these propositions were framed, namely: That the mineral acids may be relied upon as a certain means of preventing the spread of the Asiatic cholera.

FRANK W. REILLY, M.D.,

—*Illinois State Register.* Springfield, Ill., Sept. 12.

---

A CARD TO THE PUBLIC.—We have just received from Bloomfield, Iowa, a "dodger," or street hand-bill which reads as follows:

"Dr. J. E. Harper, A.M., M.D. Professor of Diseases of the Eye and Ear in the College of Physicians and Surgeons of Chicago, expects to visit me next Saturday to remain a few days. I would be pleased to meet all who have diseased eyes or ears at my office during his stay here. Respectfully,

"J. B. FINDLEY."

The style of the hand-bill can not be reproduced here, as some of the letters are two inches in length. If this kind of advertising is all right, we would be glad to know it. For information, we call upon the State Boards of Iowa and Illinois.—(*Peoria Medical Monthly*, Sept., 1883).

